# Association mapping uncovers maize *ZmbZIP107* regulating root system architecture and lead absorption under lead stress

**DOI:** 10.3389/fpls.2022.1015151

**Published:** 2022-09-26

**Authors:** Fengxia Hou, Kai Liu, Na Zhang, Chaoying Zou, Guangsheng Yuan, Shibin Gao, Minyan Zhang, Guangtang Pan, Langlang Ma, Yaou Shen

**Affiliations:** State Key Laboratory of Crop Gene Exploration and Utilization in Southwest China, Maize Research Institute, Sichuan Agricultural University, Chengdu, China

**Keywords:** maize, lead tolerance, root system architecture, GWAS, gene-based association study, ZmbZIP107

## Abstract

Lead (Pb) is a highly toxic contaminant to living organisms and the environment. Excessive Pb in soils affects crop yield and quality, thus threatening human health *via* the food chain. Herein, we investigated Pb tolerance among a maize association panel using root bushiness (BSH) under Pb treatment as an indicator. Through a genome-wide association study of relative BSH, we identified four single nucleotide polymorphisms (SNPs) and 30 candidate genes associated with Pb tolerance in maize seedlings. Transcriptome analysis showed that four of the 30 genes were differentially responsive to Pb treatment between two maize lines with contrasting Pb tolerance. Among these, the *ZmbZIP107* transcription factor was confirmed as the key gene controlling maize tolerance to Pb by using gene-based association studies. Two 5’ UTR_variants in *ZmbZIP107* affected its expression level and Pb tolerance among different maize lines. ZmbZIP107 protein was specifically targeted to the nucleus and *ZmbZIP107* mRNA showed the highest expression in maize seedling roots among different tissues. Heterologous expression of *ZmbZIP107* enhanced rice tolerance to Pb stress and decreased Pb absorption in the roots. Our study provided the basis for revelation of the molecular mechanism underlying Pb tolerance and contributed to cultivation of Pb-tolerant varieties in maize.

## Introduction

Heavy metal pollution from industrial and agricultural activities has become one of the most important environmental concerns worldwide. Heavy metals can cause irreversible damages to plants and thus lead to serious harm to human health through the food chain (Pourrut et al., 2013; Singh et al., 2015). Among various heavy metals, lead (Pb) is widespread in the environment and easily accumulated by plants through leaf adsorption or root enrichment (Gupta et al., 2013; Pourrut et al., 2013; Schreck et al., 2013). Pb exerts negative effects on plant growth and development of plants, such as inhibiting seed germination (Lamhamdi et al., 2011), root elongation (Huang et al., 2019), chlorophyll synthesis (Prasad and Prasad, 1987), cell division (Samardakiewicz and Woźny, 2005), and mineral element absorption (Seoud and Bakr, 2020), and causing membrane cell damage and thus cell death (Sharma and Dubey, 2005).

Furthermore, excessive Pb in soils seriously affect the yield and quality of plants (Alam et al., 2003; Dinakar et al., 2008). On the other hand, plants have evolved a variety of defense mechanisms to against the toxic action of Pb, including sequestration of Pb in vacuoles (Maestri et al., 2010), chelation of Pb in cytosols (Thapa et al., 2012), bind of Pb to organic acids (Thapa et al., 2012; Gupta et al., 2013), activation of antioxidant defense systems (Verma and Dubey, 2003; Gupta et al., 2009), and modification of soil rhizosphere environment (Jia et al., 2013).

Root system is a crucial vegetative organ for plant fixation, reproduction, and nutrient uptake (Beemster and Baskin, 1998). In addition, root system plays a significant role in plant growth and development, rhizosphere environment regulation and adaptation to abiotic stress through root number, root length, root surface area, root volume, and root bushiness and biomass (Grzesiak et al., 1999; Huang et al., 2019). Pb is absorbed and transported by plants mainly through their root systems, and approximately 90% of the total Pb is maintained in the roots for most plants (Kumar et al., 1995). In addition, root system responds to Pb stress by modulating root volume and diameter, such as inhibiting lateral root formation and thus reducing the volume of total roots. Under Pb stress, the viability of rice root cells was affected and cell death was promoted (Huang and Huang, 2008). The previous studies showed that Pb toxicity negatively affected *Lemna minor* root growth by inhibiting root tip cell division (Eun et al., 2000; Samardakiewicz and Woźny, 2005). Under Pb treatment, the root length and root dry weight were significantly reduced in wheat (Kaur et al., 2013), maize (Kozhevnikova et al., 2009), pea (Malecka et al., 2009), and *Sedum alfredii* (Gupta et al., 2010). Moreover, Pb treatment resulted in significantly inhibited root length and surface area in *Elsholtzia argyi* and *Elsholtzia splendens* (Peng et al., 2005). The recently developed 3D image reconstruction technology facilitates the studies on original traits of plant roots, such as root bushiness (BSH), root volume, and root diameter, relative to classical root traits (e.g. root length, root surface area, and total number of roots) (Mollier and Botany, 1999). Among these original traits, bushiness is described as ratio of the maximum of roots to the median of roots, which is considered an important indicator to evaluate the lush degree of plant roots and has been widely studied in soybeans, maize (Rangarajan and Lynch, 2021), Arabidopsis (Liang et al., 2018), and rice (Ambreetha et al., 2017).

Some genes involved in heavy metal tolerance have been reported in plants. Nature resistance-associated Macrophage Protein (NRAMP) family was previously demonstrated to play important roles in absorption and transport of heavy metals in plants. Among the NRAMP members, *OsNRAMP5* prevents the transport of cadmium to xylem and significantly reduced the cadmium (Cd) content in rice grains and shoots (Chang et al., 2020). However, heterologous expression of *OsNRAMP1* promoted Cd and arsenic accumulation in Arabidopsis (Tiwari et al., 2014). Yellow Stripe-Like (YSL) proteins are a kind of transporters encoding metal nicotinamides, which regulate metal uptake, transport, and distribution in a variety of plants. Among the YSL family, *BdYSL3*, *OsYSL16*, and *OsYSL2* modulate transport of intracellular copper, iron, and manganese (Mn) (Ishimaru et al., 2010; Zhang et al., 2018; Foroozani, 2021). Metal tolerance proteins (MTPs) are involved in metal homeostasis and tolerance in plants. Overexpression of *OsMTP8.1* enhanced Mn accumulation and tolerance in yeast and rice (Chen et al., 2013). Moreover, *OsMTP9* also participates in absorption and transport of Mn in rice (Ueno et al., 2015). In addition, several Pb stress-related genes have been reported in different plant species. Expression of *SbLRR2* in Arabidopsis conferred promoted root growth and reduced Pb accumulation in the transgenic Arabidopsis plants (Zhu et al., 2013). Expression of *AtATM3* in Indian mustard enhanced its tolerance to Pb and Cd stresses (Bhuiyan et al., 2011). *PSE1* that encodes an unknown NC domain-containing protein regulates Pb tolerance in Arabidopsis by activating expression of ABC transporter *PDR12/ABCG40* (Fan et al., 2016). In our previous study, the *ZmHIPP* gene, which encodes a heavy metal-associated isoprenylated plant protein was demonstrated to positively mediate Pb accumulation and tolerance in maize seedlings (Ma et al., 2022). Plant basic leucine zipper (bZIP) transcription factors are a relatively conserved and large gene families. A number of bZIP members have been identified in the plants, animals, and yeasts (Newman and Keating, 2003; Nam et al., 2021). Previous studies indicated that the bZIP transcription factors play significant roles as regulators in signal transduction and response to diverse biotic/abiotic stresses, such as drought, cold, heat, ABA, and salt stresses (Weltmeier et al., 2006; Hossain et al., 2010; Liu et al., 2014; Li et al., 2020b). These studies on the bZIP function mainly focused on rice, maize, soybean, tomato, wheat, Arabidopsis, and banana (Kobayashi et al., 2008; Liao et al., 2008; Nijhawan et al., 2008; Hsieh et al., 2010; He et al., 2013; Yang et al., 2013). To date, only few studies reported the involvement of the bZIP genes in heavy metal stresses in plants.

Genome-wide association study (GWAS) is widely used to identify genetic variations and candidate genes of abiotic stress response-related traits in maize. A total of 21 single nucleotide polymorphisms (SNPs) for Pb accumulation were identified in a panel of 269 maize accessions using GWAS (Zhao et al., 2018). In a maize panel consisting of 230 inbred lines, 37, 12, 13, 27, and 23 SNPs were significantly associated with mercury accumulation in the grains, axes, stems, bracts, and leaves, respectively, by using GWAS, with the phenotype variation explained (PVE) by each SNP ranging from 6.91% to 15.87% (Zhao et al., 2017). In addition, 328 SNPs associated with root traits under high/low nitrate conditions were detected in an association panel of 461 maize inbred lines, with the PVE of a single SNP varying between 5.21% and 8.81% (Xsa et al., 2020). Our previous study performed GWAS for seed chilling-germination in maize and uncovered 15 significant associations controlling seed germination under cold stress (Zhang et al., 2021). By combining gene-based association studies, four genes were further confirmed as the potential causal genes (Zhang et al., 2021). In the present study, GWAS was performed in an association panel of 312 maize inbred lines to detect SNPs and candidate genes associated with relative root BSH under Pb stress. Combined with transcriptome data derived from our previous studies, the potential causual genes were identified from these candidate genes. Candidate gene association studies further confirmed that the genetic variants in the 5’ UTR of *ZmbZIP107* affected Pb tolerance of root BSH among maize lines. Finally, we investigated the role of *ZmbZIP107 in vivo* by heterologous expression of *ZmbZIP107* in rice, which validated the effects of *ZmbZIP107* on Pb tolerance and accumulation in rice seedlings. Collectively, these results suggest that *ZmbZIP107* is a positive regulator of Pb tolerance in maize seedlings, which inhibits Pb absorption in the roots.

## Materials and methods

### Plant materials, growth conditions, and phenotypic investigations

A maize association panel of 312 diverse inbred lines, which included the Tropical, Stiff Stalk (SS), and non-Stiff Stalk (NSS) germplasms (Zhang et al., 2016a), was used to identify the genetic associations with Pb tolerance in maize seedlings. Seeds of these 312 lines were cultivated in quartz sands with a photoperiod of 16/8 h (day/night) and a temperature of 25/22 °C (Abdel-Ghani et al., 2016). At the two-leaf stage, the consistent-growth seedlings of each line were divided into two groups, with one group transplanted into a Hoagland solution supplemented with 1 mM Pb(NO_3_)_2_ (as Pb treatment, T) and the other into a Hoagland solution without Pb(NO_3_)_2_ (as control check condition, CK). pH of these two solutions were maintained at approximately 4.5 with titration of KOH or HCl when required (Cunningham, 1996; Małkowski et al., 2005; Bashmakov et al., 2017). On the 7^th^ day of culture, root bushiness (BSH) was measured for each line using an EPSON Expression 10000XL Root Scanner and assessed using the ARIA software (Pace et al., 2014). The 312 maize lines were arranged in a randomized complete block design with three replications. The BSH of each line under Pb treatment and normal conditions was represented by BSH-T and BSH-CK, respectively. The lead-tolerance coefficient (LTC) of BSH was calculated as the ratio of the BSH under Pb treatment to that under CK, which was used to evaluate maize tolerance to Pb treatment in GWAS (Ma et al., 2022).

### Statistical analysis of phenotype data

For each line, the average of BSH across three replicates was used as the phenotype of BSH. The SPSS (v. 20, IBM, N, USA) software was used for descriptive statistical analysis of phenotypic data, including mean, standard deviation (SD), skewness, kurtosis, frequency distribution, and coefficient of variation (CV).

### Genome-wide association studies and candidate gene identification

The genotypes of 312 association panel were constructed using a maize SNP chip (56K) in our previous study (Zhang et al., 2016a). The following criteria were used for SNP quality filtering in this study: minor allele frequency (MAF) < 0.05, missing rate > 0.2, and the SNPs with heterozygosity > 20% (Ma et al., 2021a). A total of 43,799 high-quality SNPs were remained for GWAS. Fixed and random model circulating probability unification (FarmCPU) model was used to detect the associations between the phenotypes and SNPs (Zhang et al., 2016a; Li et al., 2020a; Ma et al., 2021a). The simpleM program in R was used to calculate the number of effective markers (n=24876), and a stringent p threshold of 0.05/n (p = 0.05/24876 = 2.01×10^-6^) was used to detect SNPs significantly associated with BSH-LTC. Based on the B73 (RefGen V2) genome, all the genes located in the LD (Linkage disequilibrium) = 300 kb regions of these significant SNPs were excavated and identified as putative candidate genes for BSH-LTC (Zhang et al., 2020a; Ma et al., 2021b).

### Gene-based association analysis

To verify whether variants within the candidate genes affected BSH-LTC among different maize lines, 77 inbred lines randomly selected from the association panel were subjected to gene-based association analysis for each candidate gene. In detail, the genomic DNA of the 77 lines were used as the templates to amplify the gene body and its 2000 bp upstream for each gene by PCR. The B73 genome sequence (RefGen V4) was used as the reference sequence to align the amplified sequences, and the DNAMAN software (v. 5.2.2, Lynnon Bio-soft, Canada) was employed to detect insertions/deletions (InDels) and SNPs (Zhang et al., 2020b). The associations between these genetic variants and BSH-LTC were calculated based on the general linear model (GLM) in TASSEL v. 5.0 (Zhang et al., 2021). The significance threshold was set as p = 0.05 according to the previous study (Ma et al., 2022). Haploview software (http://www.broad.mit.edu/mpg/haploview) was used to calculate the LD between the markers (Hou et al., 2021). For each gene, haplotypes were identified according to these trait-associated SNPs and a *t*-test was used to analyze significance of BSH-LTC between contrasting haplotypes.

### Determination of Pb concentration

On the 14^th^ day of Pb treatment, rice seedlings were soaked in 20 mM Na_2_-EDTA and then rinsed with distilled water for three times. The roots were sampled and dried at 105°C for 2 h and 70°C to a constant weight. These samples were then digested with 10 mL nitric acid (HNO_3_) using the MARSX (CEM) microwave digester. Finally, Pb content was determined by the Inductively Coupled Plasma Mass Spectrometry (NexION 1000 ICP-MS). Pb concentration (mg/kg DW) was represented by Pb content per unit dry weight (DW).

### Real-time quantitative reverse-transcription PCR

Nine tissues were collected from maize line B73 and subjected to expression analysis of *ZmbZIP107*, including roots (three-leaf stage and silking stage), leaf (three-leaf stage and silking stage), stems (silking stage), female flowers, male flowers, grains on the 6^th^ day after pollination (6 DAP) and 12 DAP. Moreover, maize seedlings were cultured in Hoagland solutions supplemented with 1mM Pb(NO_3_)_2_ and without Pb(NO_3_)_2_ for seven days, respectively. The shoots and roots were individually sampled for total RNA extraction by using TRIzol Reagent (Invitrogen, California, USA). The first-strand cDNA was synthesized from 2 µg total RNA using a reverse transcription kit (Novoprotein, Nanjing, China) following the manufacturer’s instruction. The gene expression level of *ZmbZIP107* was quantified by performing RT-qPCR with SYBR qPCR SuperMix Plus (Novoprotein, Nanjing, China). The *OsActin* gene was used an internal reference, and the 2^-ΔΔCT^ method was used to calculate the relative expression levels of *ZmbZIP107* (Chen et al., 2021). Three biological replicates and three technical replicates were included for each sample.

### Subcellular localization of ZmbZIP107

To verify the subcellular localization of the ZmbZIP107 protein, we cloned the coding sequence (CDS) of *ZmbZIP107* without the stop codon into the *p*CAMBIA2300-*eGFP* vector, generating the *p35S:ZmbZIP107-eGFP* fusion expression vector. Then then fusion vector was transformed into *Agrobacterium tumefaciens* strain GV3101 and the transformed Agrobacterium suspension with OD_600 =_ 0.8 were injected into the *Nicotiana benthamiana* leaves for transient expression. The *p35S:eGFP* vector was used as the negative control. eGFP fluorescence signal in the transformed tobacco leaves was detected at 48 h after culture with a confocal microscope (Zeiss LSM 800, Baden-Württemberg, Germany).

### Rice transformation and phenotypic identification

The full-length CDS of *ZmbZIP107* was cloned from the favorable-haplotype line (B73) and integrated into the *p*CUB vector under the control of Ubi promoter. The recombinant plasmid was transferred into the rice cultivar ZHONG HUA 11 through Agrobacterium-mediated transformation to produce *ZmbZIP107*-overexpressed lines. Positive transgenic plants were detected by using *ZmbZIP107*-based PCR. Seeds from the transgenic and wild-type plants were incubated on the germinating paper at 37°C in the darkness. Germinated seeds were transferred to a germinating box filled with distilled water and cultured for seven days. Then, the seedlings with uniform growth were transferred to nutrient solutions (pH = 4.5) with 50 mg/L Pb(NO_3_)_2_ and without Pb(NO_3_)_2_, respectively (Chen et al., 2007). These rice plants were then grown in a culture room with a photoperiod of 14/10 h (day/night) and a temperature of 28/25 °C for 14 days (Nam et al., 2021). Shoot length (SHL) were measured using a ruler. The following root traits were measured using EPSON expression 10000XL root scanner: BSH, total root length (TRL), total surface area (TSA), primary root length (PRL), secondary root length (SEL), and maximum number of roots (MNR).

## Results

### Statistical descriptions of BSH under CK and T conditions

**Figure 1:**
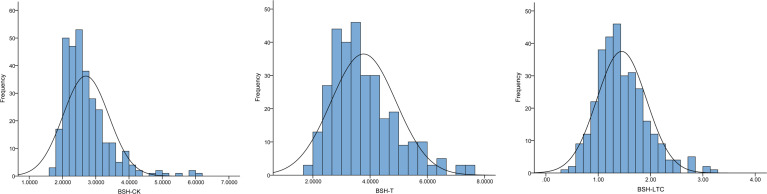
Frequency distribution of BSH under different conditions. BSH, root bushiness; CK, normal condition; T, Pb treatment; LTC, lead-tolerance coefficient.

To evaluate the natural variations of BSH, we investigated BSH under normal and Pb treatment conditions in a maize association panel composed of 312 inbred lines. The phenotypic frequency of BSH displayed a normal distribution under both conditions among this panel (**Figure 1**). The mean values of BSH were 2.70 and 3.77 under CK and T conditions with the range from 1.67 to 6.00 and 1.67 to 7.51, respectively, showing a significant difference (p < 0.001) between the two conditions (**Table 1**). The CVs of BSH were 25.56% (CK) and 30.24% (T) (**Table 1**), implying that Pb treatment improved the phenotypic variation of BSH among different lines. Furthermore, we calculated the lead-tolerance coefficient (LTC) of BSH to evaluate Pb tolerance for each line. The averaged BSH-LTC was 1.45 among the association panel, with the range from 0.33–3.26 (**Table 1**), suggesting that Pb treatment had a generally positive effect on BSH of this panel.

**Table 1:** Phenotypic variations of BSH in the maize association panel.

Trait	Range	Mean	SD	Skewness	Kurtosis	CV (%)	Sig.
BSH-CK	1.67-6.00	2.70	0.69	1.89	5.32	25.56	***
BSH-T	1.67-7.51	3.77	1.14	0.92	0.71	30.24	
BSH-LTC	0.33-3.26	1.45	0.47	0.86	1.27	32.41	-

BSH, Bushiness; LTC, lead-tolerance coefficient; CK, normal condition; T, Pb treatment; SD, standard deviation; CV, coefficient of variation; *** significant at p < 0.001 level between BSH-CK and BSH-T.

### Genetic loci and candidate genes controlling BSH-LTC

**Figure 2:**
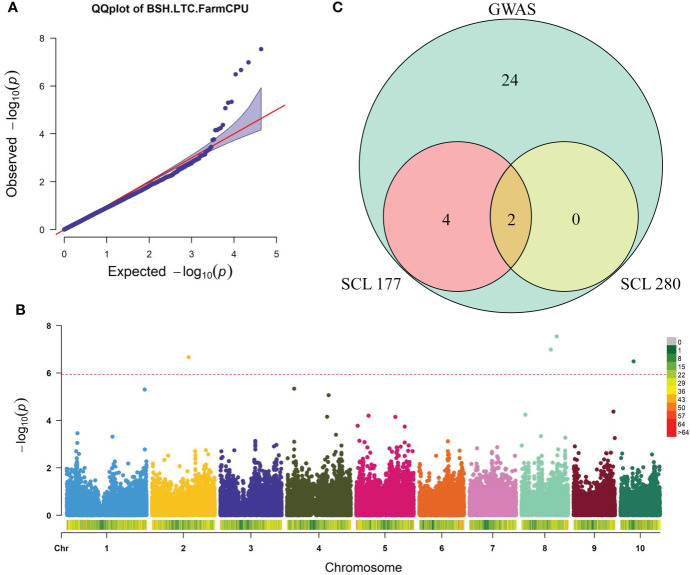
Combined genome-wide association study (GWAS) and transcriptome data revealing potential causal genes of maize tolerance to Pb stress. **(A, B)** Quantile-quantile (Q-Q) plot **(A)** and Manhattan plot **(B)** of the GWAS for BSH-LTC by the FarmCPU model. BSH, root bushiness; LTC, lead-tolerance coefficient. The dashed red line shows the significance threshold (2.01×10^-6^) **(C)** Wayne diagram displaying the Pb treatment-responsive genes among 30 candidate genes identified by GWAS. SCL177, a Pb-sensitive maize line; SCL280, a Pb-tolerant maize line.

To explore the genetic architecture of BSH-LTC in maize, we performed a GWAS for BSH-LTC using the FarmCPU model with 43,799 high-quality SNPs. The quantile-quantile plot (Q-Q plot) showed a sharp deviation from the expected p value specially in the tail region (**Figure 2A**), suggesting that FarmCPU did not result in many false positives in our GWAS. Finally, four SNPs were significantly associated with BSH-LTC at a stringent p threshold of 2.01 × 10^−6^ (0.05/24876) (**Figure 2B**). These significant SNPs were located on chromosomes 2, 8 and 10, with p value ranging from 3.24×10^-7^ (PZE-110032152) to 2.87×10^-8^ (PZE-108077298). The PVE of each SNP varied between 1.89% (PZE-108062009) and 4.80% (PZE-108077298) (**Figure 2B**, **Table 2**), implicating that BSH-LTC in maize were mainly controlled be several minor loci. According to our previous studies, the linkage disequilibrium (LD) decay distance of this panel was approximately 300 kb. Accordingly, a total of 30 gene models were identified in the 300 kb flanking regions of the four BSH-LTC-associated SNPs (**Table S1**).

**Table 2:** Significant SNPs associated with BSH-LTC identified by GWAS using FarmCPU model.

Trait	SNP	Chr	Alleles	Position (bp)	P-value	PVE (%)
BSH-LTC	PZE-108077298	8	C/A	132854286	2.87E-08	4.80
	PZE-108062009	8	A/G	110849647	1.03E-07	1.89
	PZE-102108114	2	A/C	137985405	2.15E-07	4.03
	PZE-110032152	10	C/G	49992422	3.24E-07	2.19

BSH, root bushiness; LTC, lead-tolerance coefficient; Chr, chromosome; SNP, single nucleotide polymorphism.

### Integration of transcriptome data to identify causal genes for Pb tolerance in maize

To further select out the key genes of BSH-LTC from the 30 candidate genes identified by GWAS, we analyzed expression patterns of each gene in response to Pb stress by using the transcriptome data. Our previous study performed transcriptome sequencing for the roots of a Pb-sensitive line (SCL177) and a Pb-tolerant line (SCL280) during the response to Pb stress (Ma et al., 2022). Finally, relative to CK conditions, six candidate genes were differentially expressed under Pb treatment in at least one line, with |log2 Ratio (48 h/0 h)| > 1 and FDR < 0.05 (**Table S2**). Among the six genes, *Zm00001d011012* and *Zm00001d007827* were differentially expressed under Pb treatment in both lines (**Figure 2C**). While *Zm00001d024160*, *Zm00001d011134*, *Zm00001d010404*, and *Zm00001d040356* were specifically differentially expressed in SCL177 under Pb stress (**Figure 2C**), which were considered the genes differentially responding to Pb treatment between the two contrasting lines (**Figure 2C**). These four genes were thereby considered the potential causal genes responsible for maize tolerance to Pb stress. According to the B73 RefGen_V4 reference genome (http://www.gramene.org), *Zm00001d024160*was annotated as a TGA4 transcription factor (ZmbZIP107) whereas *Zm00001d011134* encodes aputative AP2/EREBP transcription factor superfamily protein (*WRI1*). *Zm00001d010404* and*Zm00001d040356* encode a putative cyclin superfamily protein and geranylgeranyl hydrogenase 2, respectively (**Table S2**).

### Variations in ZmbZIP107 affecting BSH-LTC among different maize lines

**Figure 3:**
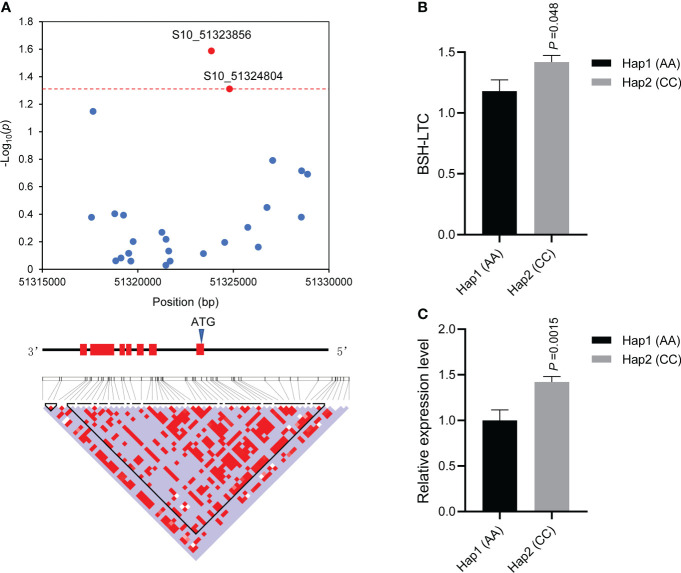
Association analysis of the candidate gene *ZmbZIP107*. **(A)** The SNPs (S10_51323856 and S10_51324804) significantly (p < 0.05) associated with BSH-LTC. The dashed red line shows the significance threshold (p = 0.05). The gene simulation structure is shown in the middle and the red filled boxes represent the exons of *ZmbZIP107*. The bottom image indicates the pairwise LDs (linkage disequilibriums) between the SNP markers. BSH, root bushiness; LTC, lead-tolerance coefficient. **(B)** Comparison of BSH-LTC between the two haplotypes, Hap1 (AA, n=12) and Hap2 (CC, n=46). **(C)** Comparison of *ZmbZIP107* expression level between the two haplotypes under Pb treatment.

To verify whether genetic variants in the above four genes influenced Pb tolerance among diverse lines, we conducted gene-based association studies for each gene. Using PCR, we amplified and sequenced the gene body and 2000 bp upstream of the four genes. Though variant calling, a total of 22 (21 SNPs and 1 InDel), 8 (7 SNPs and 1 InDel), 25 (25 SNPs), and 5 (5 SNPs) polymorphisms were identified in *Zm00001d010404*, *Zm00001d040356*, *Zm00001d024160*, and *Zm00001d011134*, respectively (**Table S3**). Association studies indicated that two SNPs (S8_113171481,C/G and S8_113172367, C/T) located in *Zm00001d010404* were significantly (p < 0.05) associated with BSH-LTC, which were situated in the seventh intron (**Figure S1A**). One SNP (S3_39647996, G/A) located in *Zm00001d040356* was significantly (p< 0.05) associated with BSH-LTC, which resided in the first exon and led to a synonymous mutation (**Figure S1B**). Moreover, one SNP (S8_140016556, C/A) located in the 5` UTR of *Zm00001d011134* was significantly (p < 0.05) associated with BSH-LTC, nevertheless, no significant difference in BSH-LTC value was observed between the lines with allele C and those with allele A (**Figures S1C, D**). Remarkably, two SNPs (S10_51323856, C/A and S10_51324804, C/A) located in the 5` UTR of *Zm00001d024160* (*ZmbZIP107*) were significantly (p< 0.05) associated with BSH-LTC (**Figure 3A**). The two significant SNPs divided the 77 lines into two major haplotypes (Hap1: AA; Hap2: CC), a *t*-test showed that the BSH-LTC (1.178) of Hap1 was significantly (p< 0.05) lower than that (1.417) of Hap 2 (**Figure 3B**). In contrast to synonymous variants and intron_variants, those in the 5’ UTR tend to cause changes in gene transcriptional levels and thus lead to phenotypic variations (Bashirullah et al., 2001; Zhang et al., 2016b). We then detected the expression of *ZmbZIP107* in the two contrasting haplotypes under Pb treatment by using RT-qPCR. The results showed that the expression level of *ZmbZIP107* in Hap1 (AA) was significantly (p< 0.01) lower than that in Hap2 (CC) under Pb treatment (**Figure 3C**). Collectively, these results suggested that the two 5` UTR_variants in *ZmbZIP107* regulated its expression level and thus influenced BSH-LTC among different maize lines. Therefore, *ZmbZIP107* was confirmed as the key gene for maize tolerance to Pb treatment in the present study.

### Spatial expression pattern of *ZmbZIP107*

**Figure 4:**
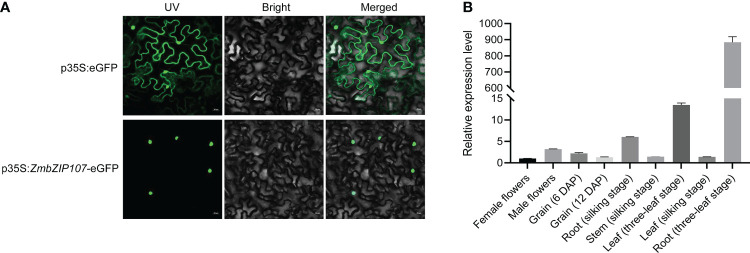
Spatial expression pattern of *ZmbZIP107*. **(A)** Subcellular localization of the ZmbZIP107 protein in tobacco leaves. (Scale bar, 20 μm). **(B)** *ZmbZIP107* expression level in different tissues of maize.

To detect subcellular localization of the ZmbZIP107 protein, we fused *ZmbZIP107* with *eGFP* and transformed it into tobacco leaves. The eGFP fluorescence signal was specifically observed in the nucleus of the leaves transformed with the *p35S:ZmbZIP107-eGFP* fusion expression vector (**Figure 4A**). In contrast, the fluorescence signal was observed in both the nucleus and cytoplasm of the leaves transformed with the *p35S:eGFP* vector (**Figure 4A**). These results showed that *ZmbZIP107* protein was specifically localized in the nucleus. Furthermore, *ZmbZIP107* expression level was detected in different maize tissues using RT-qPCR. The results displayed that *ZmbZIP107* showed the highest expression in the roots (three-leaf stage), followed by the leaves (three-leaf stage) and roots (silking stage). However, the female flower had the lowest expression level of *ZmbZIP107*. These findings supported the role of *ZmbZIP107* in mediating root system architecture (**Figure 4B**).

### Heterologous expression of *ZmbZIP107* enhancing Pb tolerance in rice seedlings

**Figure 5:**
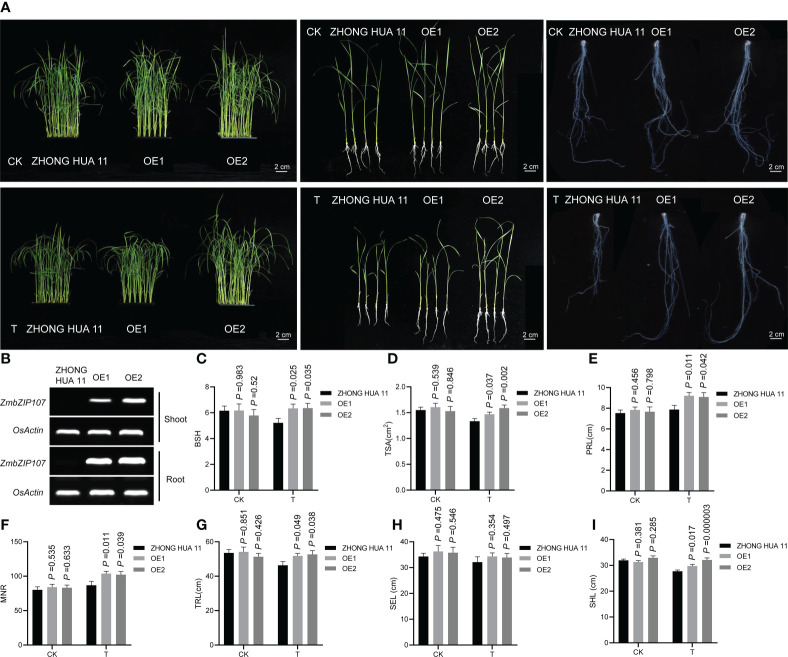
Heterologous expression of *ZmbZIP107* improving lead tolerance in rice. **(A)** Phenotypic comparison between ZHONG HUA 11 and the overexpression lines (OE1 and OE2) under CK and T conditions. CK, normal condition; T, Pb treatment, (Scale bar, 2 cm). **(B)** Expression of *ZmbZIP107* in the roots and shoots of the OE lines under Pb treatment, detected using semi-quantitative RT-PCR. **(C–I)** Root system architecture (RSA) of ZHONG HUA 11 and the OE lines under CK and T conditions. CK, normal condition; T, Pb treatment. BSH, root bushiness; TSA, total surface area; PRL, primary root length; SEL, secondary root length; MNR, maximum number of roots; TRL, total root length; SHL, Shoot length.

To investigate the regulatory role of *ZmbZIP107* in Pb tolerance, we obtained two independent rice transgenic lines (OE1 and OE2) overexpressing *ZmbZIP107* with the favorable haplotype (Hap2) (**Figure 5A**). Semi-quantitative RT-PCR showed that *ZmbZIP107* was efficiently expressed in both roots and shoots of OE1 and OE2, with higher expression levels in OE2 than those in OE1 (**Figure 5B**). We then compared the BSH between the OE lines and wild type under Pb and CK conditions. Under CK conditions, no significant difference in BSH was observed between each of the OE lines and wild type (**Figure 5C**). Under Pb treatment, the BSH was significantly (p< 0.05) increased by 21.5% (OE1) and 21.9% (OE2) relative to the wild type (**Figure 5C**). Meanwhile, the BSH-LTC values of these OE lines were 1.026 (OE1) and 1.098 (OE2), which were higher than that (0.846) of the wild type. To further test the overexpressed *ZmbZIP107* influenced the root system architecture under Pb stress, we investigated five root architecture-related traits (TRL, TSA, PRL, SEL, and MNR) and shoot length (SHL) in the OE lines and ZHONG HUA 11 under normal conditions and Pb treatment. Ultimately, *ZmbZIP107* overexpression had no significant effect on TRL, TSA, PRL, SEL, MNR, and SHL in the early vegetative stage of rice under normal conditions (**Figures 5D-I**). Notably, compared with those in ZHONG HUA 11, the phenotypes of four root architecture traits (TRL, TSA, PRL, and MNR) and SHL were significantly (p < 0.05) increased in the two OE lines under Pb stress, with increasing average percentage varying between 7.3% (SHL) and 19.5% (MNR) (**Figures 5D-I**). Moreover, a higher SHL was observed in OE2 than that in OE1, which was probably owing to a higher *ZmbZIP107* overexpression level in OE2 than that in OE1. However, the overexpressed *ZmbZIP107* did not affect SEL of rice under Pb treatment **(** **Figure 5H** **)**. Combined these results suggested that heterologous expression of *ZmbZIP107* enhanced Pb tolerance of rice roots and shoots.

### Heterologous expression of *ZmbZIP107* decreasing Pb uptake in rice roots

**Figure 6:**
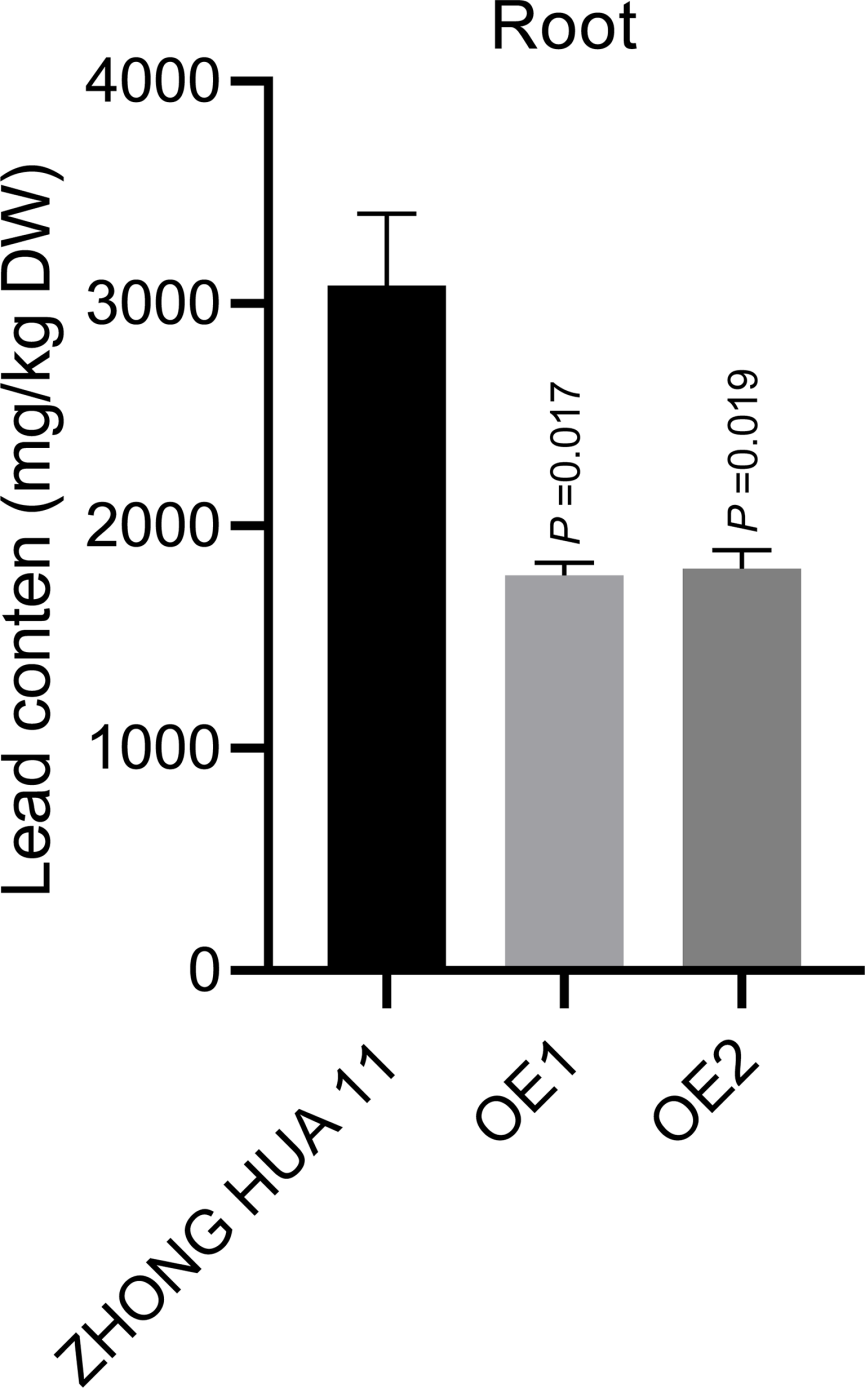
Pb content in the roots of *ZmbZIP107*-overexpressed lines (OE1 and OE2) and ZHONG HUA 11. DW represents dry weight of roots.

To investigate the effect of *ZmbZIP107* on Pb accumulation, we measured Pb content in the roots of the *ZmbZIP107*-OE lines and ZHONG HUA 11 under the condition of 50 mg/L Pb treatment. The results showed that Pb concentration in these OE lines was significantly decreased by 42.3% (OE1) and 41.4% (OE2) relative to the wild-type ZHONG HUA 11 under Pb treatment (**Figure 6**). These suggested that increased *ZmbZIP107* expression in rice transgenic lines effectively inhibited Pb absorption by the roots, alleviating Pb toxicity and improving root and shoot growth under Pb stress.

## Discussions

### Using BSH-LTC as an indicator of maize seedlings tolerance to Pb stress

Plant roots play an important role in plant growth including anchoring plants in the soil, absorbing water and nutrients, and providing the site for the rhizosphere to interact with beneficial organisms. Meanwhile, root system is the foremost organ of direct contact with heavy metal ions in the environment (Lu et al., 2013). Pb toxicity to plant roots mainly involves rapid inhibition of root growth (Godbold and Kettner, 1991; Gzyl et al., 1997), and the symptom of Pb toxicity in plant roots is the result of restriction in cell division (Przymusiński and Woźny, 1985; Wierzbicka and Botany, 1989; Wozny and Plantarum, 1991). Reduction in root length and biomass caused by Pb stress has been reported in various plants, including tomato (Van der Merwe et al., 2009), rice (Xiao-Ming et al., 2009), maize (Kozhevnikova et al., 2009), and wheat (Kaur et al., 2013). Root length of rice seedlings was reduced by 40% under the treatment of 0.5–1.0 mM Pb^2+^ (Verma and Dubey, 2003). Peng et al. showed that the root length and surface area were significantly inhibited under Pb stress in Elsholtzia argyi and Elsholtzia splendens (Peng et al., 2005). Pb stress also inhibited lateral root formation by affecting root diameter and volume (Hou et al., 2021). Currently, root dry weight has thereby been widely considered a reliable indicator for evaluating plant tolerance to abiotic stresses during early growth stages (Pour-Aboughadareh et al., 2020; Ma et al., 2022).

Root bushiness that is equal to the ratio of the maximum of roots to the median of roots has been extensively used to evaluate the lush degree of plant roots in different species (Ambreetha et al., 2017; Liang et al., 2018; Rangarajan and Lynch, 2021). Herein, for the first time, we used BSH-LTC that reflects the relative BSH under Pb treatment as an indicator for evaluating maize seedlings tolerance to Pb stress. The CV of BSH-LTC was 32.41% among the maize panel and even exceeded BSH under CK conditions (25.56%) and Pb treatment (30.24%) (**Table 1**), suggesting that BSH-LTC can comprehensively reflect the variations of Pb tolerance-related traits among different lines. Moreover, the broad-sense heritability of BSH-LTC in maize was > 30%, which was similar to those of other root traits in maize (Pace et al., 2014). It suggested that BSH-LTC was significantly affected by genetic factors and suitable for phenotyping the maize panel in GWAS. Finally, overexpression of the *ZmbZIP107* gene that was identified by using BSH-LTC caused increased BSH-LTC in rice, verifying the reliability of employing BSH-LTC to evaluate Pb tolerance.

### Combining GWAS and gene-based association analysis to identify causal gene of maize tolerance to Pb stress

GWAS is an effective method to dissect the genetic basis of quantitative traits. It has been widely applied to identify a variety of complex traits in plant species, including Pb accumulation (Zhao et al., 2018), protein abundance (Jiang et al., 2021), root configuration-related traits (Wang et al., 2019), yield-related traits (Sharma et al., 2018), salt tolerance (Ma et al., 2021b), and so on. Gene-based association analysis is the most widespread method for associating the functional variation loci of candidate genes with the phenotypes they produce (Li et al., 2019). In soybean, a combination of GWAS and candidate gene association study identified a phosphorus efficiency-related gene *GmACP1* and a functional marker Indel170 (Zhang et al., 2014). Similarly, using this strategy, an InDel located in the *ZmDREB2.7* promoter was found to confer maize resistance to drought stress (Liu et al., 2013). Moreover, Chao et al. used 149 Arabidopsis lines to perform candidate gene association analysis for a *Heavy Metal Associated* gene (*AtHMA3*), uncovering five SNPs acting on Cd accumulation in maize leaves (Chao et al., 2012).

In the present study, four significant SNPs were identified using GWAS (**Table 2**), and a total of 30 gene models were located in the LD regions of these loci (**Table S1**). Among the 30 genes, four were differentially responsive to Pb treatment between two maize lines with contrasting Pb tolerance, which were thus considered the potential causal genes for Pb tolerance (**Table S2**). To further identify the key gene, we performed gene-based association studies for each of the four genes. Finally, only two variants in the 5` UTR of *ZmbZIP107* were significantly associated with BSH-LTC (**Figure 3**). In summary, we selected out a causal gene (*ZmbZIP107*) from the GWAS results by combining transcriptome analysis and gene-based association mapping, which suggested the high efficiency of using the integration strategy in excavating functional genes. On the other hand, the identified favorable haplotype in *ZmbZIP107* can be used for developing functional markers in molecular marker-assisted selection.

### Application of *ZmbZIP107* in breeding Pb-tolerant varieties and soil phytoremediation

Increased heavy metal concentration in soils has caused serious health threats for plants, animals, and human beings. It is vitally significant to remediate soil environment and cultivate plant varieties tolerant to heavy metals. In our study, we cloned a transcription factor *ZmbZIP107* that was responsible for Pb tolerance and accumulation in the roots of maize seedlings. The heterologous expression of *ZmbZIP107* enhanced Pb tolerance and inhibited Pb absorption in rice. In the future, using transgenic technologies to increase *ZmbZIP107* expression in other crops is desirable to improve Pb tolerance of germplasm resources and cultivate varieties with low Pb accumulation. As a widely grown crop with large biomass, maize has a great potential of soil bioremediation (Ma et al., 2022). CRISPR/Cas9 and RNAi can be used for generating *ZmbZIP107*-knockout and knockdown maize lines, respectively, breeding maize varieties with high Pb accumulation for soil phytoremediation.

## Data availability statement

The original contributions presented in the study are publicly available. This data can be found here: https://ngdc.cncb.ac.cn/, CRA004789

## Author contributions

YS conceived the study. YS and LM designed the experiments. FH, KL, and NZ conducted the experiments. FH, CZ, GY, and MZ performed the data analysis. GP and SG provide the maize panel. FH and YS drafted the manuscript. All authors contributed to the article and approved the submitted version.

## Funding

This work is supported by the National Key Research and Development Program of China (2021YFF1000303), the Sichuan Science and Technology Program (2021JDTD0004 and 2021YJ0476), and the earmarked fund for China Agriculture Research System (CARS-02).

## Conflict of interest

The authors declare that the research was conducted in the absence of any commercial or financial relationships that could be construed as a potential conflict of interest.

## Publisher’s note

All claims expressed in this article are solely those of the authors and do not necessarily represent those of their affiliated organizations, or those of the publisher, the editors and the reviewers. Any product that may be evaluated in this article, or claim that may be made by its manufacturer, is not guaranteed or endorsed by the publisher.

## References

[B1] Abdel-Ghani A. H. Sanchez D. L. Kumar B. Lubberstedt T. (2016). Paper roll culture and assessment of maize root parameters. Bio-protocol 6 (18), e1926. doi: 10.21769/Bioprotoc.1926

[B2] Alam M. G. Snow E. T. Tanaka A. (2003). Arsenic and heavy metal contamination of vegetables grown in samta village, Bangladesh. Sci. Total Environ. 308 (1-3), 83–96. doi: 10.1016/S0048-9697(02)00651-4 12738203

[B3] Ambreetha S. Chinnadurai C. Marimuthu P. Balachandar D. J. R. (2017). Plant-associated bacillus modulates the expression of auxin-responsive genes of rice and modifies the root architecture. Rhizosphere 5, 57–66. doi: 10.1016/j.rhisph.2017.12.001

[B4] Bashirullah A. Cooperstock R. L. Lipshitz H. D. (2001). Spatial and temporal control of RNA stability. Proc. Natl. Acad. Sci. U. S. A. 98 (13), 7025–7028. doi: 10.1073/pnas.111145698 11416182 PMC34617

[B5] Bashmakov D. I. Kluchagina A. N. Malec P. Strzałka K. Lukatkin A. S. (2017). Lead accumulation and distribution in maize seedlings: Relevance to biomass production and metal phytoextraction. Int. J. Phytoremediation 19 (11), 1059–1064. doi: 10.1080/15226514.2017.1319334 28441031

[B6] Beemster G. T. Baskin T. I. (1998). Analysis of cell division and elongation underlying the developmental acceleration of root growth in arabidopsis thaliana. Plant Physiol. 116 (4), 1515–1526. doi: 10.1104/pp.116.4.1515 9536070 PMC35060

[B7] Bhuiyan M. Min S. R. Jeong W. J. Sultana S. Choi K. S. Lee Y. . (2011). Overexpression of AtATM3 in brassica juncea confers enhanced heavy metal tolerance and accumulation. Plant Cell Tissue Organ Culture (PCTOC). 107 (1), 69–77. doi: 10.1007/s11240-011-9958-y

[B8] Chang J. D. Huang S. Konishi N. Wang P. Chen J. Huang X. Y. . (2020). Overexpression of the manganese/cadmium transporter OsNRAMP5 reduces cadmium accumulation in rice grain. J. Exp. botany. 71, 5705–5715. doi: 10.1093/jxb/eraa287 32542348

[B9] Chao D. Y. Silva A. Baxter I. Huang Y. S. Nordborg M. Danku J. . (2012). Genome-wide association studies identify heavy metal ATPase3 as the primary determinant of natural variation in leaf cadmium in arabidopsis thaliana. PloS Genet. 8 (9), e1002923. doi: 10.1371/journal.pgen.1002923 22969436 PMC3435251

[B10] Chen H. Fang R. Deng R. Li J. (2021). The OsmiRNA166b-OsHox32 pair regulates mechanical strength of rice plants by modulating cell wall biosynthesis. Plant Biotechnol. J. 19 (7), 1468–1480. doi: 10.1111/pbi.13565 33560572 PMC8313131

[B11] Chen Z. Fujii Y. Yamaji N. Masuda S. Takemoto Y. Kamiya T. . (2013). Mn Tolerance in rice is mediated by MTP8.1, a member of the cation diffusion facilitator family. J. Exp. botany. 64 (14), 4375–4387. doi: 10.1093/jxb/ert243 23963678 PMC3808320

[B12] Chen J. Zhu C. Li L. P. Sun Z. Y. Pan X. B. (2007). Effects of exogenous salicylic acid on growth and H2O2-metabolizing enzymes in rice seedlings under lead stress. J. Environ. Sci. 19 (1), 44–49. doi: 10.1016/s1001-0742(07)60007-2 17913152

[B13] Cunningham J. J. N. P. (1996). Lead phytoextraction: Species variation in lead uptake and translocation. New Phytologist 134 (1), 75–84. doi: 10.2307/2558516

[B14] Dinakar N. Nagajyothi P. C. Suresh S. Udaykiran Y. Damodharam T. (2008). Phytotoxicity of cadmium on protein, proline and antioxidant enzyme activities in growing arachis hypogaea l. seedlings. J. Environ. Sci. 20 (2), 199–206. doi: 10.1016/s1001-0742(08)60032-7 18574962

[B15] Eun S. O. Youn H. S. Lee Y. (2000). Lead disturbs microtubule organization in the root meristem of zea mays. Physiologia Plantarum 110 (3), 357–65. doi: 10.1111/j.1399-3054.2000.1100310.x

[B16] Fan T. Yang L. Wu X. Ni J. Jiang H. Zhang Q. . (2016). The PSE1 gene modulates lead tolerance in arabidopsis. J. Exp. botany. 67 (15), 4685–4695. doi: 10.1093/jxb/erw251 27335453 PMC4973742

[B17] Foroozani M. (2021). The role of the yellow stripe-like transporter BdYSL3 in copper homeostasis in brachypodium. Plant Physiol. 186 (1), 204–205. doi: 10.1093/plphys/kiab092 33772275 PMC8154040

[B18] Godbold D. L. Kettner C. (1991). Use of root elongation studies to determine aluminium and lead toxicity in picea abies seedlings. J. Plant Physiol. 138 (2), 231–235. doi: 10.1016/S0176-1617(11)80276-2

[B19] Grzesiak S. Hura T. Grzesiak M. T. Plantarum A.P (1999). The impact of limited soil moisture and waterlogging stress conditions on morphological and anatomical root traits in maize (Zea mays l.) hybrids of different drought tolerance. Acta Physiologiae Plantarum 21 (3), 305–315. doi: 10.1007/s11738-999-0046-4

[B20] Gupta D. K. Huang H. G. Corpas F. J. (2013). Lead tolerance in plants: Strategies for phytoremediation. Environ. Sci. pollut. Res. Int. 20 (4), 2150–2161. doi: 10.1007/s11356-013-1485-4 23338995

[B21] Gupta D. K. Huang H. G. Yang X. E. Razafindrabe B. H. N. Inouhe M. (2010). The detoxification of lead in sedum alfredii h. @ is not related to phytochelatins but the glutathione. J. hazardous materials. 177 (1-3), 437–444. doi: 10.1016/j.jhazmat.2009.12.052 20047791

[B22] Gupta D. K. Nicoloso F. T. Schetinger M. R. Rossato L. V. Pereira L. B. Castro G. Y. . (2009). Antioxidant defense mechanism in hydroponically grown zea mays seedlings under moderate lead stress. J. hazardous materials. 172 (1), 479–484. doi: 10.1016/j.jhazmat.2009.06.141 19625122

[B23] Gzyl J. Przymusinski R. Wozny A. J. A. S. B. P. (1997). Organospecific reactions of yellow lupin seedlings to lead. Acta Societatis Botanicorum Poloniae 66 (1), 61–66. doi: 10.5586/asbp.1997.009

[B24] He S. Shan W. Kuang J. F. Xie H. Xiao Y. Y. Lu W. J. . (2013). Molecular characterization of a stress-response bZIP transcription factor in banana. Plant Cell Tissue Organ Culture 113, 173–187. doi: 10.1007/s11240-012-0258-y

[B25] Hossain M. A. Cho J. I. Han M. Ahn C. H. Jeon J. S. An G. . (2010). The ABRE-binding bZIP transcription factor OsABF2 is a positive regulator of abiotic stress and ABA signaling in rice. J. Plant Physiol. 167 (17), 1512–1520. doi: 10.1016/j.jplph.2010.05.008 20576316

[B26] Hou F. Zhou X. Liu P. Yuan G. Zou C. Lübberstedt T. . (2021). Genetic dissection of maize seedling traits in an IBM Syn10 DH population under the combined stress of lead and cadmium. Mol. Genet. Genomics 296 (5), 1057–1070. doi: 10.1007/s00438-021-01800-2 34117523

[B27] Hsieh T. H. Li C. W. Su R. C. Cheng C. P. Sanjaya Tsai Y. C. . (2010). A tomato bZIP transcription factor, SlAREB, is involved in water deficit and salt stress response. Planta 231 (6), 1459–1473. doi: 10.1007/s00425-010-1147-4 20358223

[B28] Huang L. Chen D. Zhang H. Song Y. Chen H. Tang M. (2019). Funneliformis mosseae enhances root development and Pb phytostabilization in robinia pseudoacacia in Pb-contaminated soil. Front. Microbiol. 10. doi: 10.3389/fmicb.2019.02591 PMC6861453 31781076

[B29] Huang T. L. Huang H. J. J. C. (2008). ROS and CDPK-like kinase-mediated activation of MAP kinase in rice roots exposed to lead. Chemosphere 71 (7), 1377–1385. doi: 10.1016/j.chemosphere.2007.11.031 18164745

[B30] Ishimaru Y. Masuda H. Bashir K. Inoue H. Tsukamoto T. Takahashi M. . (2010). Rice metal-nicotianamine transporter, OsYSL2, is required for the long-distance transport of iron and manganese. Plant J. 62 (3), 379–390. doi: 10.1111/j.1365-313X.2010.04158.x 20128878

[B31] Jia X. Dong S. Zhou C. (2013). Responses of microorganism in the rhizosphere of winter wheat seedlings to a low concentration of lead. Advance J. Food Sci. Tech. 5 (5), 633–639. doi: 10.19026/ajfst.5.3139. J.A.J.o.F.S., and Technology.

[B32] Jiang L. Wang Y. Xia A. Wang Q. Zhang X. Jez J. M. . (2021). A natural single-nucleotide polymorphism variant in sulfite reductase influences sulfur assimilation in maize. New phytologist 232 (2), 692–704. doi: 10.1111/nph.17616 34254312

[B33] Kaur G. Singh H. P. Batish D. R. Kohli R. K. (2013). Lead (Pb)-induced biochemical and ultrastructural changes in wheat (Triticum aestivum) roots. Protoplasma 250 (1), 53–62. doi: 10.1007/s00709-011-0372-4 22231903

[B34] Kobayashi F. Maeta E. Terashima A. Takumi S. (2008). Positive role of a wheat HvABI5 ortholog in abiotic stress response of seedlings. Physiologia plantarum. 134 (1), 74–86. doi: 10.1111/j.1399-3054.2008.01107.x 18433415

[B35] Kozhevnikova A. D. Seregin I. V. Bystrova E. I. Belyaeva A. I. Kataeva M. N. Ivanov V. B. (2009). The effects of lead, nickel, and strontium nitrates on cell division and elongation in maize roots. Russian J. Plant Physiol. 56 (2), 242–250. doi: 10.1134/S1021443709020137

[B36] Kumar P. B. A. N. Dushenkov V. Motto H. Raskin I. J. (1995). Phytoextraction: The use of plants to remove heavy metals from soils. Environ. Sci. And Tech. 29 (5), 1232–1238. doi: 10.1021/es00005a014. E.S., and Technology. 22192016

[B37] Lamhamdi M. Bakrim A. Aarab A. Lafont R. Sayah F. (2011). Lead phytotoxicity on wheat (Triticum aestivum l.) seed germination and seedlings growth. Comptes rendus biologies. 334 (2), 118–126. doi: 10.1016/j.crvi.2010.12.006 21333942

[B38] Liang Y. Zhao X. Jones A. M. Gao Y. J. P. S. (2018). G Proteins sculp root architecture in response to nitrogen in rice and arabidopsis. Plant Science. 274, 129–136. doi: 10.1016/j.plantsci.2018.05.019 30080596

[B39] Liao Y. Zhang J. S. Chen S. Y. Zhang W. K. (2008). Role of soybean GmbZIP132 under abscisic acid and salt stresses. J. Integr. Plant Biol. 50 (2), 221–230. doi: 10.1111/j.1744-7909.2007.00593.x 18713445

[B40] Li N. Lin B. Wang H. Li X. Yang F. Ding X. . (2019). Natural variation in ZmFBL41 confers banded leaf and sheath blight resistance in maize. Nat. Genet. 51 (10), 1540–1548. doi: 10.1038/s41588-019-0503-y 31570888

[B41] Li Z. Liu P. Zhang X. Zhang Y. Ma L. Liu M. . (2020a). Genome-wide association studies and QTL mapping uncover the genetic architecture of ear tip-barrenness in maize. Physiologia plantarum. 170 (1), 27–39. doi: 10.1111/ppl.13087 32175598

[B42] Li Z. Tang J. Srivastava R. Bassham D. C. Howell S. H. (2020b). The transcription factor bZIP60 links the unfolded protein response to the heat stress response in maize. Plant Cell. 32 (11), 3559–3575. doi: 10.1105/tpc.20.00260 32843434 PMC7610289

[B43] Liu C. Mao B. Ou S. Wang W. Liu L. Wu Y. . (2014). OsbZIP71, a bZIP transcription factor, confers salinity and drought tolerance in rice. Plant Mol. Biol. 84 (1-2), 19–36. doi: 10.1007/s11103-013-0115-3 23918260

[B44] Liu S. Wang X. Wang H. Xin H. Yang X. Yan J. . (2013). Genome-wide analysis of ZmDREB genes and their association with natural variation in drought tolerance at seedling stage of zea mays l. PloS Genet. 9, e1003790. doi: 10.1371/journal.pgen.1003790 24086146 PMC3784558

[B45] Lu Z. Zhang Z. Su Y. Liu C. Shi G. (2013). Cultivar variation in morphological response of peanut roots to cadmium stress and its relation to cadmium accumulation. Ecotoxicol. Environ. safety. 91, 147–155. doi: 10.1016/j.ecoenv.2013.01.017 23410837

[B46] Ma L. An R. Jiang L. Zhang C. Li Z. Zou C. . (2022). Effects of ZmHIPP on lead tolerance in maize seedlings: Novel ideas for soil bioremediation. J. hazardous materials. 430, 128457. doi: 10.1016/j.jhazmat.2022.128457 35180524

[B47] Maestri E. Marmiroli M. Visioli G. Marmiroli N. J. E. Botany E. (2010). Metal tolerance and hyperaccumulation: Costs and trade-offs between traits and environment. Environ. Exp. Botany. 68 (1), 1–13. doi: 10.1016/j.envexpbot.2009.10.011

[B48] Malecka A. Piechalak A. Tomaszewska B. (2009). Reactive oxygen species production and antioxidative defense system in pea root tissues treated with lead ions: the whole roots level. Acta Physiologiae Plantarum. 31 (5), 1053–1063. doi: 10.1007/s11738-009-0326-z

[B49] Małkowski E. Kurtyka R. Kita A. Karcz W. (2005). Accumulation of Pb and cd and its effect on Ca distribution in maize seedlings [Zea mays l.]. Polish J. Environ. Stud. 14 (2), 203–207.

[B50] Ma L. Qing C. Zhang M. Zou C. Pan G. Shen Y. (2021a). GWAS with a PCA uncovers candidate genes for accumulations of microelements in maize seedlings. Physiologia plantarum. 172 (4), 2170–2180. doi: 10.1111/ppl.13466 34028036

[B51] Ma L. Zhang M. Chen J. Qing C. He S. Zou C. . (2021b). GWAS and WGCNA uncover hub genes controlling salt tolerance in maize (Zea mays l.) seedlings. Theor. Appl. Genet. 134 (10), 3305–3318. doi: 10.1007/s00122-021-03897-w 34218289

[B52] Mollier Botany P. (1999). Maize root system growth and development as influenced by phosphorus deficiency. J. Exp. Botany. 50 (333), 487–497. doi: 10.1016/S0142-9612(01)00350-7

[B53] Nam H. I. Shahzad Z. Dorone Y. Clowez S. Zhao K. Bouain N. . (2021). Interdependent iron and phosphorus availability controls photosynthesis through retrograde signaling. Nat. Commun. 12 (1), 7211. doi: 10.1038/s41467-021-27548-2 34893639 PMC8664907

[B54] Newman J. R. Keating A. E. (2003). Comprehensive identification of human bZIP interactions with coiled-coil arrays. Science 300 (5628), 2097–2101. doi: 10.1126/science.1084648 12805554

[B55] Nijhawan A. Jain M. Tyagi A. K. Khurana J. P. (2008). Genomic survey and gene expression analysis of the basic leucine zipper transcription factor family in rice. Plant Physiol. 146 (2), 333–350. doi: 10.1104/pp.107.112821 18065552 PMC2245831

[B56] Pace J. Lee N. Naik H. S. Ganapathysubramanian B. Lübberstedt T. (2014). Analysis of maize (Zea mays l.) seedling roots with the high-throughput image analysis tool ARIA (Automatic root image analysis). PloS One 9 (9), e108255. doi: 10.1371/journal.pone.0108255 25251072 PMC4176968

[B57] Peng H. Y. Tian S. K. Yang X. E. (2005). Changes of root morphology and Pb uptake by two species of elsholtzia under Pb toxicity. J. Zhejiang Univ. Sci. B. 6 (6), 546–552. doi: 10.1631/jzus.2005.B0546 15909342 PMC1389888

[B58] Pour-Aboughadareh A. Etminan A. Abdelrahman M. Tran L. S. P. Siddique K.H.M.J.P.G.R. (2020). Assessment of biochemical and physiological parameters of durum wheat genotypes at the seedling stage during polyethylene glycol-induced water stress. Plant Growth Regulation 92 (1), 81–93. doi: 10.1007/s10725-020-00621-4

[B59] Pourrut B. Shahid M. Douay F. Dumat C. Pinelli E. (2013). Molecular mechanisms involved in lead uptake, toxicity and detoxification in higher plants. Heavy Metal Stress in Plants 292, 121–147. doi: 10.1007/978-3-642-38469-1_7

[B60] Prasad D. Prasad A. J. P. (1987). Effect of lead and mercury on chlorophyll synthesis in mung bean seedlings. Phytochemistry 26 (4), 881–883. doi: 10.1016/S0031-9422(00)82310-9

[B61] Przymusiński R. Woźny A. (1985). The reactions of lupin roots on the presence of lead in the medium. Biochemie Und Physiologie Der Pflanzen. 180 (4), 309–318. doi: 10.1016/S0015-3796(85)80007-X

[B62] Rangarajan H. Lynch J. P. (2021). A comparative analysis of quantitative metrics of root architecture. Plant Phenomics. 2021, 6953197. doi: 10.34133/2021/6953197 33851135 PMC8028844

[B63] Samardakiewicz S. Woźny A. (2005). Cell division in lemna minor roots treated with lead. Aquat. Botany. 83 (4), 289–295. doi: 10.1016/j.aquabot.2005.06.007

[B64] Schreck E. Laplanche C. Le Guédard M. Bessoule J. J. Austruy A. Xiong T. . (2013). Influence of fine process particles enriched with metals and metalloids on lactuca sativa l. leaf fatty acid composition following air and/or soil-plant field exposure. Environ. pollution 179, 242–249. doi: 10.1016/j.envpol.2013.04.024 23694728

[B65] Seoud I. I. A. E. Bakr E. M. A. (2020). Alleviation of cadmium and lead stress by a-mycorrhizal fungi and rock phosphate on maize (Zea mays l.) growth under calcareous soil conditions. Environ. Qual. Manage. 30 (2), 75–89. doi: 10.1002/tqem.21713

[B66] Sharma R. Draicchio F. Bull H. Herzig P. Maurer A. Pillen K. . (2018). Genome-wide association of yield traits in a nested association mapping population of barley reveals new gene diversity for future breeding. J. Exp. botany. 69 (16), 3811–3822. doi: 10.1093/jxb/ery178 29767798 PMC6054221

[B67] Sharma P. Dubey R. S. (2005). Lead toxicity in plants. Brazilian. J. Plant Physiol. 17 (1), , 491–500. doi: 10.1515/9783110434330-015

[B68] Singh S. Parihar P. Singh R. Singh V. P. Prasad S. M. (2015). Heavy metal tolerance in plants: Role of transcriptomics, proteomics, metabolomics, and ionomics. Front. Plant science 6. doi: 10.3389/fpls.2015.01143 PMC4744854 26904030

[B69] Thapa G. Sadhukhan A. Panda S. K. Sahoo L. (2012). Molecular mechanistic model of plant heavy metal tolerance. biometals: An international journal on the role of metal ions in biology, biochemistry, and medicine. Biometals 25 (3), 489–505. doi: 10.1007/s10534-012-9541-y 22481367

[B70] Tiwari M. Sharma D. Dwivedi S. Singh M. Tripathi R. D. Trivedi P. K. (2014). Expression in arabidopsis and cellular localization reveal involvement of rice NRAMP, OsNRAMP1, in arsenic transport and tolerance. Plant Cell environment 37 (1), 140–152. doi: 10.1111/pce.12138 23700971

[B71] Ueno D. Sasaki A. Yamaji N. Miyaji T. Fujii Y. Takemoto Y. . (2015). A polarly localized transporter for efficient manganese uptake in rice. Nat. plants 1, 15170. doi: 10.1038/nplants.2015.170 27251715

[B72] Van der Merwe M. J. Osorio S. Moritz T. Nunes-Nesi A. Fernie A. R. (2009). Decreased mitochondrial activities of malate dehydrogenase and fumarase in tomato lead to altered root growth and architecture *via* diverse mechanisms. Plant Physiol. 149 (2), 653–669. doi: 10.1104/pp.108.130518 19028880 PMC2633863

[B73] Verma S. Dubey R. (2003). Lead toxicity induces lipid peroxidation and alters the activities of antioxidant enzymes in growing rice plants. Plant ence. 164 (4), 645–655. doi: 10.1016/S0168-9452(03)00022-0

[B74] Wang H. Wei J. Li P. Wang Y. Ge Z. Qian J. . (2019). Integrating GWAS and gene expression analysis identifies candidate genes for root morphology traits in maize at the seedling stage. Genes 10 (10), 773. doi: 10.3390/genes10100773 PMC6826382 31581635

[B75] Weltmeier F. Ehlert A. Mayer C. S. Dietrich K. Wang X. Schütze K. . (2006). Combinatorial control of arabidopsis proline dehydrogenase transcription by specific heterodimerisation of bZIP transcription factors. EMBO J. 25 (13), 3133–3143. doi: 10.1038/sj.emboj.7601206 16810321 PMC1500977

[B76] Wierzbicka M. J. E. Botany E. (1989). Disturbances in cytokinesis caused by inorganic lead. Environ. Exp. Botany. 29 (2), 123–133. doi: 10.1016/0098-8472(89)90044-0

[B77] Wozny A. Plantarum E. J. J. B. (1991). The effect of lead on early stages ofPhaseolus vulgaris l. growth*in vitro* conditions. Biol. Plantarum. 33 (1), 32–39. doi: 10.1007/BF02873785

[B78] Xiao-Ming L. I. Yin Y. L. Huang Y. J. He-Bao X. U. J. S. (2009). Effects of lead pollution in soil and on leaf on distribution and accumulation of lead in rice. Soils 41 (4), 556–61. doi: 10.13758/j.cnki.tr.2009.04.005

[B79] Xsa B. Wei R. A. Peng W. A. Fc A. Ly A. Qp A. . (2020). Evaluation of maize root growth and genome-wide association studies of root traits in response to low nitrogen supply at seedling emergence - ScienceDirect. Crop J. 9 (4), 794–804. doi: 10.1016/j.cj.2020.09.011

[B80] Yang Y. G. Lv W. T. Li M. J. Wang B. Sun D. M. Deng X. (2013). Maize membrane-bound transcription factor Zmbzip17 is a key regulator in the cross-talk of ER quality control and ABA signaling. Plant Cell Physiol. 54 (12), 2020–2033. doi: 10.1093/pcp/pct142 24092882

[B81] Zhang X. Guan Z. Li Z. Liu P. Ma L. Zhang Y. . (2020a). A combination of linkage mapping and GWAS brings new elements on the genetic basis of yield-related traits in maize across multiple environments. Theor. Appl. Genet. 133 (10), 2881–2895. doi: 10.1007/s00122-020-03639-4 32594266

[B82] Zhang Y. Hu Y. Guan Z. Liu P. He Y. Zou C. . (2020b). Combined linkage mapping and association analysis reveals genetic control of maize kernel moisture content. Physiologia plantarum. 170 (4), 508–518. doi: 10.1111/ppl.13180 32754968

[B83] Zhang Y. Liu P. Wang C. Zhang N. Zhu Y. Zou C. . (2021). Genome-wide association study uncovers new genetic loci and candidate genes underlying seed chilling-germination in maize. PeerJ 9, e11707. doi: 10.7717/peerj.11707 34249517 PMC8247712

[B84] Zhang C. Lu W. Yang Y. Shen Z. Ma J. F. Zheng L. (2018). OsYSL16 is required for preferential Cu distribution to floral organs in rice. Plant Cell Physiol. 59 (10), 2039–2051. doi: 10.1093/pcp/pcy124 29939322

[B85] Zhang D. Song H. Cheng H. Hao D. Wang H. Kan G. . (2014). The acid phosphatase-encoding gene GmACP1 contributes to soybean tolerance to low-phosphorus stress. PloS Genet. 10 (1), e1004061. doi: 10.1371/journal.pgen.1004061 24391523 PMC3879153

[B86] Zhang X. Zhang H. Li L. Lan H. Ren Z. Liu D. . (2016a). Characterizing the population structure and genetic diversity of maize breeding germplasm in southwest China using genome-wide SNP markers. BMC Genomics 17 (1), 697. doi: 10.1186/s12864-016-3041-3 27581193 PMC5007717

[B87] Zhang X. M. Zha G. M. Wang J. Wang X. J. Song S. Shu J. C. . (2016b). Comparation of the effects of different 5’-untranslated regions (UTRs) on gene expression in HEK293 cells. Biotechnol. letters 38 (12), 2051–2057. doi: 10.1007/s10529-016-2199-8 27580891

[B88] Zhao Z. Fu Z. Lin Y. Chen H. Liu K. Xing X. . (2017). Genome-wide association analysis identifies loci governing mercury accumulation in maize. Sci. Rep. 7 (1), 247. doi: 10.1038/s41598-017-00189-6 28325924 PMC5427852

[B89] Zhao X. Liu Y. Wu W. Li Y. Luo L. Lan Y. . (2018). Genome-wide association analysis of lead accumulation in maize. Mol. Genet. Genomics 293 (3), 615–622. doi: 10.1007/s00438-017-1411-4 29274071

[B90] Zhu F. Y. Li L. Lam P. Y. Chen M. X. Chye M. L. Lo C. (2013). Sorghum extracellular leucine-rich repeat protein SbLRR2 mediates lead tolerance in transgenic arabidopsis. Plant Cell Physiol. 54 (9), 1549–1559. doi: 10.1093/pcp/pct101 23877877

