# Supplementary figures and images for: Association mapping uncovers maize ZmbZIP107 regulating root system architecture and lead absorption under lead stress
Source: Front Plant Sci. 2022 Sep 26;13:1015151. doi: 10.3389/fpls.2022.1015151 (PMC9549328; doi:10.3389/fpls.2022.1015151)

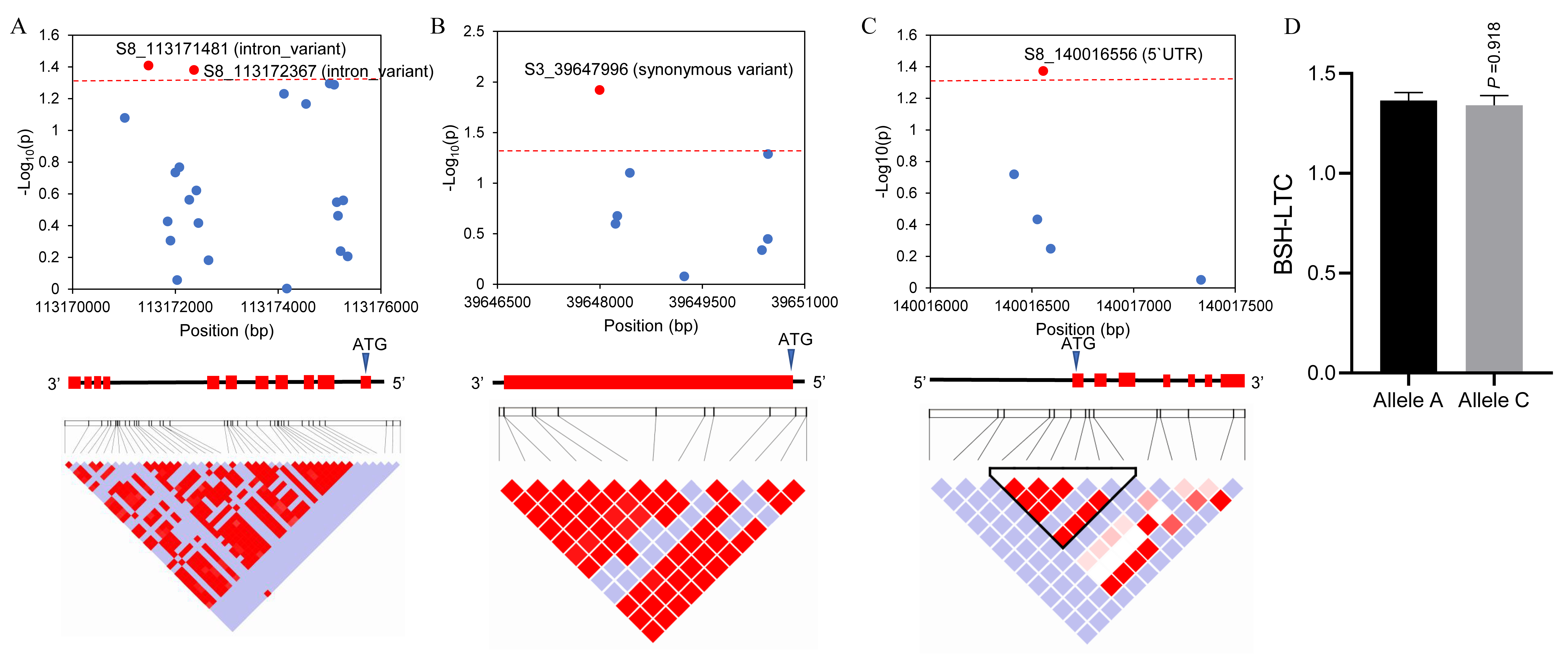

Supplement: Supplementary file 4 [file Image_1.tif]
